# Use and barriers to the use of telehealth services among the Arab population in Israel

**DOI:** 10.1093/eurpub/ckac131.181

**Published:** 2022-10-25

**Authors:** M Laron, N Penn

**Affiliations:** Health Policy Team, Myers JDC Brookdale, Jerusalem, Israel

## Abstract

**Background:**

Telehealth services tends to be used relatively infrequently by minority populations, thereby exacerbating health inequalities. This study examines the individual, circumstantial and environmental factors that facilitate or hinder usage of telehealth among Israeli Arabs, who constitute 21% of the Israeli population.

**Methods:**

Data was collected through a telephone survey among the adult Arab population in October 2020 with 501 respondents (42% response rate). Analysis included logistic regression.

**Results:**

Most of the Arab population use the internet several times a week (93%) and have a smartphone (96%). The most popular telehealth service was telephone appointments with a doctor (66%). Two thirds have never used the health plan’s mobile application, though most have no objection to using chat (75%) or video conversation (51%) with a medical professional. The most significant barrier to using telehealth is lack of awareness of services such as ordering medicines (23%). Conversely, factors that facilitate the use of telehealth include previous acquaintance with the doctor (91%); Arabic services (82%); and recommendation by health professionals (79%). Multivariate analyses indicate a strong positive correlation between education and the use of telehealth for written correspondence with a known health professional (p = 0.001).

**Conclusions:**

Telehealth services (e.g., phone appointments) which are already used widely by the Israeli-Arab population, should be retained and developed further. In parallel, digital health literacy and linguistically and culturally adaptation of digital services should be promoted. Awareness of those services should be enhanced through culturally adapted marketing and via recommendations from the family doctor.

**Key messages:**

• Identification of the barriers to the use of telehealth services among minority populations can help service providers reduce usage gaps between minority and majority population.

• The use of telehealth services should be simplified to suit people with a low digital health literacy.

